# The relationship between postpartum negative life events and postpartum depression: a moderated mediation model of neuroticism and psychological flexibility

**DOI:** 10.1186/s12888-024-05594-6

**Published:** 2024-02-21

**Authors:** Ningning Hu, Jie Luo, Wei Xiang, Guiying Yang, Ting Huang, Li Guan, Jufang Xu, Biao Peng

**Affiliations:** 1 School of Nursing, Xinjiang Medical University, Urumqi, China; 2 School of Psychology, Guizhou Normal University, Guiyang, China; 3 School of Marxism, Changsha University, Changsha, China; 4 The First People’s Hospital of Liangshan Yi Autonomous Prefecture, Xichang, China; 5 Chishui people’s hospital, Zunyi, China; 6 Hainan women and Children’s Medical Center, Haikou, China; 7 The Second Affiliated Hospital of Xinjiang Medical University, Urumqi, China; 8 School of Marxism, Changsha Social Work College, Changsha, China

**Keywords:** Postpartum negative life events, Neuroticism, Postpartum depression, Psychological flexibility

## Abstract

**Background:**

Postpartum depression (PPD) is a major public health problem worldwide. Previous studies have shown that postpartum negative life events and neuroticism are both important risk factors for PPD. However, few studies have considered the role of protective factors in the influence of postpartum negative life events and neuroticism on PPD. Based on the diathesis–stress model and Acceptance and Commitment Therapy (ACT), a moderated mediating model was established to examine the mediating role of neuroticism between postpartum negative life events and PPD, as well as the moderating role of psychological flexibility in this mediating effect.

**Methods:**

A sample of 776 parturients from three different Grade A hospitals in China were assessed using the Edinburgh Postpartum Depression Scale, the Postpartum Negative Life Events Scale, the Neuroticism Subscale of the Big Five Personality Scale, and the Acceptance and Action Questionnaire– II.

**Results:**

PPD, postpartum negative life events, neuroticism, and experiential avoidance were significantly positively correlated with one another. Neuroticism partially mediated the relationship between postpartum negative life events and PPD. In this mediation model, the direct path and the second half of the mediation path were moderated by psychological flexibility. Specifically, the links between postpartum negative life events and PPD, as well as between neuroticism and PPD, were stronger when psychological flexibility was low, but weaker when psychological flexibility was high.

**Conclusions:**

The results show that psychological flexibility plays an important role in buffering the negative effects of postpartum negative life events and neuroticism on PPD. These findings provide implications for the prevention and intervention of PPD using an ACT approach.

## Introduction

Postpartum depression (PPD) is the most common symptom of maternal mental disorders, and its symptoms include sadness, despair, low self-evaluation, depression, inability to feel happiness with one’s baby, anxiety, loss of appetite, poor attention and memory, sleep disorders, long-term fatigue, social isolation, and even suicidal thoughts and, in severe cases, thoughts of harming one’s baby [1]. PPD symptoms can last up for up to 10 days and often resolve on their own. However, 13–20% of new mothers experience a longer duration of these symptoms, and as a result, can develop PPD [2]. World Health Organization research has shown that the incidence of depression in women after childbirth is three times higher than that in other periods of their life [3]. A meta-analysis covering the years 2000 to 2021 showed that the global prevalence of PPD was 17.22% [4]. In the Chinese maternal population, the total detection rate of PPD was 15% (covering the years 2001 to 2019) [5]. PPD among Chinese women has attracted the attention of the Chinese government which, in the Healthy China Initiative (2019–2030), clearly calls for action to promote better mental health, as well as improved maternal and child health, so as to prevent and intervene against PPD [6]. Given this, exploring the influencing mechanism of PPD in Chinese women will allow for better understandings and empirical support for the development of interventions or prevention measures to counter incidences of PPD.

### Postpartum negative life events and PPD

The occurrence of PPD usually involves multi-dimensional risk factors which can be biological, psychological, or related to the social environment [7, 8]. As a social environmental factor, postpartum negative life events have attracted much attention from researchers for their universality and uncontrollability. Postpartum negative life events refer to the daily life events related to childbirth, child-rearing, and life adjustment, but with specific consideration of maternal difficulties in coping with these within the first year after delivery [9]. According to the diathesis–stress model, as a source of stress, postpartum negative life events can trigger the onset of depression and other mental disorders [10]. Cognitive behavioral therapy explains this process accordingly: when mothers are faced with the stressor of postpartum negative life events and cannot cope effectively, dysfunctional beliefs become activated, generating more biased interpretations, negative thoughts, and emotional experiences, which then leads to depression [11]. Meta-analysis has shown that negative postpartum life events such as high life pressure, lack of social support, abuse, marital conflict, delivery and postpartum physical conditions, newborn conditions, and postpartum sleep problems are all important risk factors for PPD [12, 13]. A longitudinal study showed that postpartum negative life events have a strong impact on PPD in Chinese parturients [14]. Overall, the predictive effect of postpartum negative life events on PPD has been confirmed by many studies, however few of these studies have focused on the mediating and moderating factors which exist between them. Therefore, it is important to explore the mediating and regulating mechanisms between postpartum negative life events and PPD in order to provide more effective guidance for the governments and other relevant institutions in the prevention of and intervention against PPD.

### The mediating role of neuroticism

According to the diathesis–stress model, diathesis (or vulnerability) is a risk factor for mental disorders as well as a source of stress (as an internal stress source), and stress can cause diseases through the degree of one’s vulnerability [10, 15]. In other words, vulnerability can not only be a direct cause of depression, it can also mediate or mediate the relationship between stressors and depression. At the same time, the predisposition model of depression holds that neuroticism and other adverse personality characteristics are important vulnerability factors in the generation and development of depression [16].

Neuroticism is a personality trait referring one’s tendency to experience negative emotions (e.g., anger, sadness, anxiety, worry, hostility, etc.) manifested according to individual differences in negative emotional responses to threats, setbacks, or losses [17]. Highly neurotic individuals have processing bias towards stimuli, negative information in particular. They have a tendency to adopt a more negative perspective and think that stimuli are threatening, thus exhibiting quicker, stronger emotional reactions in response, and are thus more prone to depression [18, 19]. Previous studies have confirmed that neuroticism is an important risk factor for increasing postpartum major depression [20, 21], and of all personality traits, neuroticism is the strongest predictor of PPD [22]. In short, neuroticism and negative life events are antecedent variables of PPD. However, existing findings have not been consistent regarding how the two variables relate to each other in the development of depression. Some studies have shown that neuroticism plays a mediating role between negative life events and depression [23, 24], while other studies have also shown that neuroticism plays a moderating role between negative life events and depression [25].

The dynamic predisposition model provides us with a theoretical explanation for this. Specifically, it proposes that the predisposition model can be extended to identify personality changes, and that negative life events will not only affect the onset of depression, but also affect one’s level of trait vulnerability [26, 27]. In other words, personality traits susceptible to depression, while relatively stable, are also influenced by negative life events. Obviously, neuroticism as a personality trait, its stability and change will be affected by environmental factors. Empirical studies have confirmed that life events are positively correlated with neuroticism, and that stressful life events and experiences can predict neuroticism [28]. Longitudinal studies have shown that neuroticism interacts with life events, that life events and experiences explain about half of the variance in neuroticism, and that life events have both short- and long-term effects on neuroticism [29]. This is explained by the mixed model, in which individual changes in neuroticism are subdivided into transitory and persistent [30]. Based on the above theories and empirical studies, then, we hypothesized that neuroticism plays a mediating role in the relationship between postpartum negative life events and PPD.

### The moderating role of psychological flexibility

The diathesis-stress model includes protective factors as well as stress and diathesis [15]. Protective factors can help individuals cope more effectively with the effects of risk factors, reducing the probability of negative outcomes for those predisposed to them [15, 31]. In other words, protective factors can moderate the negative effects that postpartum negative life events (stressors) and neuroticism (predisposition traits) could have on individuals. According to Acceptance and Commitment Therapy (ACT), psychological flexibility is one such protective factor.

Psychological flexibility refers to one’s ability to consciously perceive and accept all experiences and take action in the moment guided by their own values [32], and is a cornerstone of mental health. It can lead to continuous and effective interventions and intervention components (e.g., acceptance, mindfulness, values, acceptance in action) which can regulate mental health and improve overall functioning [33]. According to ACT, when faced with stressors, individuals with low psychological flexibility (i.e., high psychological inflexibility) attach themselves to a conceptualized self, maintain inflexible attention to the present moment, and are unable to take meaningful action, thus enhancing the influence of stressors on PPD [33]. Individuals with high psychological flexibility are able to maintain their self-awareness, holding flexible attention to the present moment, and can take meaningful actions to deal with stressors, thus, alleviating the influence of stressors on PPD to a far greater extent [34]. Among the effects of neuroticism on PPD, individuals with low psychological flexibility tend to adopt experiential avoidance to deal with negative experiences (e.g., negative body feelings, thoughts, memories, emotions, etc.) brought about by neuroticism. Although experiential avoidance can alleviate these negative experiences in the short term, the frequency and intensity of negative experiences will be higher in the long term, which can sustain or even exacerbate depression [35]. Individuals with high psychological flexibility, meanwhile, adopt acceptance to deal with the various negative experiences brought about by neuroticism, and are actively able to accept their own feelings, thoughts, and emotions, and objectively perceive their physical self, thus relieving the risk of depression caused by negative experiences [35].

Previous studies have confirmed that a higher level of psychological flexibility protects women from depression symptoms when faced with high-intensity stress [36], and that a lack of psychological flexibility is one mechanism for the persistence of PPD symptoms [37]. In summary, we hypothesized that psychological flexibility moderates both the direct pathway to the mediating model (i.e., the effect of postpartum negative life events on PPD) as well as the second half of the mediating effect (i.e., the effect of neuroticism on PPD).

### The current study

**Fig. 1:**
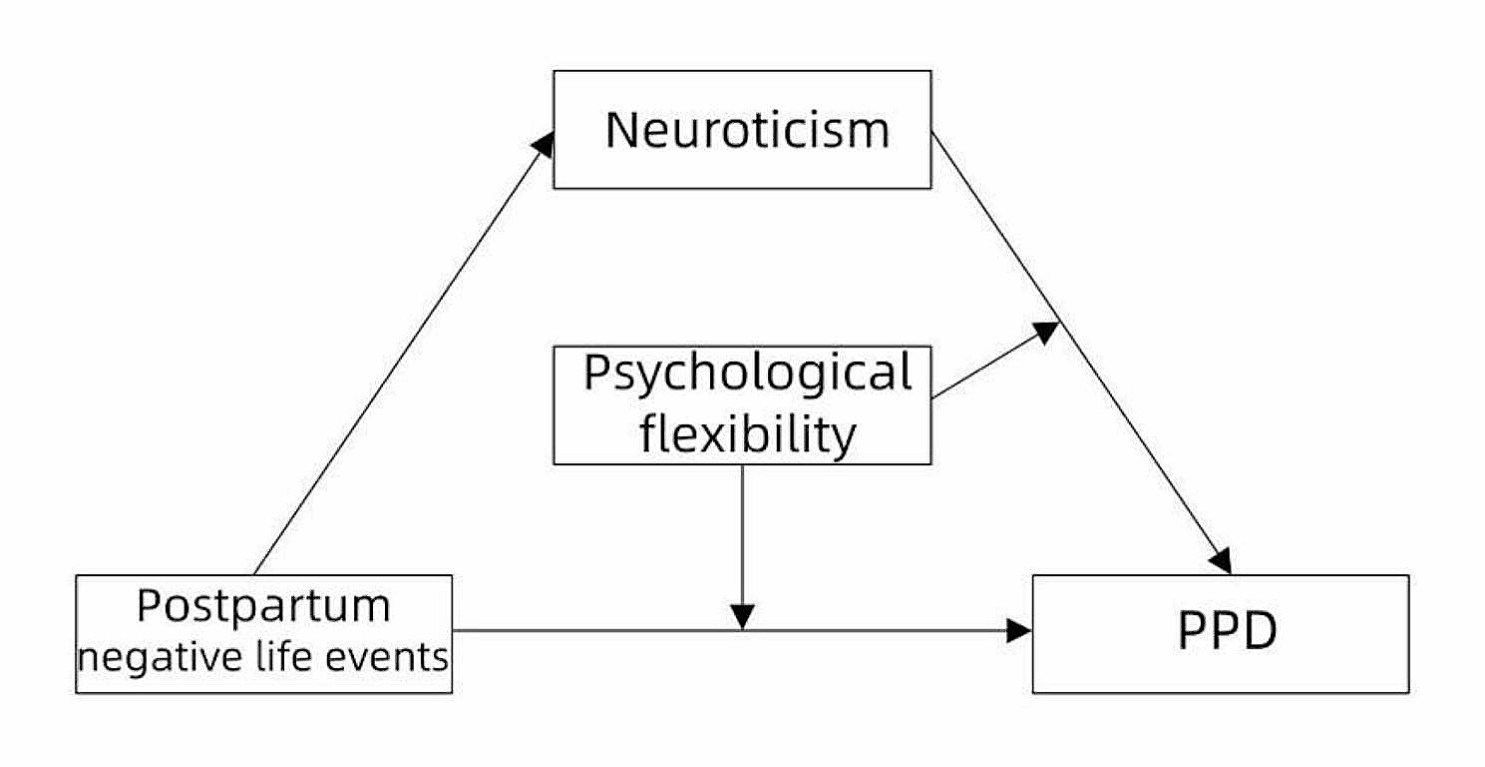
Hypotheses model of the association between postpartum negative life events and postpartum depression (PPD)

Although the relationship between negative life events and depression has already been confirmed by a large number of studies, and the mediating role of neuroticism between negative life events and depression has also been verified in college students and adolescents specifically [23, 24], few studies have verified this mediating role in parturients. More importantly, few studies have considered the moderating effect of protective factors in this mediation model. To the best of our knowledge, we are the first to investigate the moderating role of psychological flexibility in the above mediation model. We therefore established a theoretical hypothesis model (as shown in Fig. 1) and proposed the following three hypotheses:

#### Hypothesis 1

Postpartum negative life events have a positive predictive effect on PPD.

#### Hypothesis 2

Neuroticism plays a mediating role between postpartum negative life events and PPD.

#### Hypothesis 3

Psychological flexibility plays a moderating role in this mediation model. Specifically, psychological flexibility plays a moderating role in both the direct pathway (i.e., the effect of postpartum negative events on PPD) as well as in the second half of the mediating pathway (i.e., the effect of neuroticism on PPD).

## Methods

### Participants and procedure

Convenience sampling was used to gather a sample of parturients who were visiting the children’s health department for their infant’s physical examination at one of three Grade A hospitals in Guizhou, Xinjiang, or Hainan provinces in China. Inclusion criteria: age ≥ 20 years old; within one year of child delivery (according to previous studies, sufferers of PPD are generally within one year post-delivery [38]); basic reading comprehension skills; no history of mental illness. All mothers gave their informed consent before completing the measures used in the study. A total of 855 questionnaires were distributed, with 776 valid questionnaires obtained after invalid questionnaires had been excluded. The Ethics Committee of The second affiliated hospital of Xinjiang medical university approved this study, and all hospitals agreed to the collection of data within their premises.

### Measures

#### Postpartum depression (PPD)

The Edinburgh postpartum depression scale (EPDS) compiled by Cox was adopted [39], specifically the Chinese version of the scale as translated by Lee et al. (1998) [40], which has been shown to have good reliability and validity. The scale includes 10 items measuring mood, self-blame, fun, anxiety, fear, coping ability, insomnia, crying, sadness, and self-injury. Each item is rated on a four-point scale, with nine points in total typically used as the critical value for identifying patients with PPD, and 12 points being the critical value for identifying patients with severe PPD. The Cronbach’s α coefficient for the scale in this study was 0.87.

### Postpartum negative life events

The postpartum negative life events scale compiled by Wen (2014) was adopted [9], which measures seven factors using 37 items. Each item is rated on a five-point scale. The higher the total score, the more serious the respondent’s negative life events. The seven factors are: lack of social support, postpartum complications, obstruction of normal life, financial pressure, marital discord, lack of parenting experience, and lack of family support. The scale has been shown to have good reliability and validity to comprehensively evaluate various stressors faced by mothers [41]. The Cronbach’s α coefficient of the scale in this study was 0.95.

### Neuroticism

The neuroticism subscale of the short version of the Big Five Personality scale, compiled by Costa et al. [42], was adopted, which has been proved to have good reliability and validity in Chinese samples [43]. The scale uses 12 items, each one rated on a five-point scale. The Cronbach’s α coefficient of scale in this study was 0.84.

### Psychological flexibility

The acceptance and action questionnaire– second edition (AAQ-II), compiled by Bond et al. [44], was adopted, specifically the Chinese version which was translated into Chinese by Cao et al. (2013) [45]. In recent years, some researchers have pointed out that AAQ-II has some shortcomings in measuring experiential avoidance, but it is still the most commonly used self-report instrument to assess the efficacy of ACT intervention [46]. Especially in the context of Chinese culture, apart from AAQ-II Chinese version, few instruments for measuring experiential avoidance have been translated into Chinese and tested for reliability and validity. Based on the extensive use of the AAQ-II Chinese version in Chinese culture, we still use AAQ-II to measure experiential avoidance. The questionnaire consists of seven items, each of which is rated using a seven-point scale. The total score of the questionnaire reflects the respondent’s psychological flexibility by measuring their experiential avoidance. The higher the total score, the higher the respondent’s level of experiential avoidance and the lower their level of psychological flexibility. The lower the total score of the questionnaire, the lower their level of experiential avoidance and the higher their level of psychological flexibility [34]. In this study, the Cronbach’s α coefficient was 0.93.

### Statistical analyses

SPSS 25.0 was used for statistical analysis of the data. The bootstrapping method was used to estimate the effect, generating 95% bias-corrected confidence intervals (CI) using 5,000 samples to determine whether the effect was significant. Significance was achieved when the 95% CI did not include zero. First, we conducted a common method bias test. Second, descriptive statistics and Pearson correlation analysis were conducted for all four variables, and standardized processing was carried out. Third, Hayes’ (2013) SPSS-process 2.15 macro (Model 4) was used to test the mediating effect [47]. Fourth, Model 14 was used to test the moderated mediation effect. Fifth, a simple slope test (Model 1) was used to analyze the moderating effect. Previous studies have shown that there are significant differences in PPD depending on age and education level among Chinese parturients [48, 49]. Therefore, age and education level were used as covariates in this study.

## Results

### Common method bias testing

The Harman single factor method was used to test the common method deviation. A total of 15 common factors with eigenvalues greater than 1 were proposed by unrotated exploratory factor analysis for all measurement items. The first common factor explained 27.27% of the total variation, which is below the 40% threshold, indicating no significant common method bias.

### Descriptive statistics

The average age of the mothers in this study was 29.92 years old (*SD* = 4.89), with 61.98% (*n* = 481) having had natural births, and 38.02% (*n* = 295) cesarean births. The mothers’ education levels were as follows: primary school 3.35% (*n* = 26), middle school 13.92% (*n* = 108), high school or technical secondary school 17.91% (*n* = 139), junior college 27.45% (*n* = 213), bachelor degree or higher 37.37% (*n* = 290). The Type of puerpera were as follows: primiparaes 45.49% (*n* = 353), multiparas 54.51% (*n* = 423). This information is shown in Table 1.

**Table 1:** Socio-demographic characteristics of participants (*N* = 776)

Characteristic		Mean (SD) / N(%)
Age		29.92 (4.89)
Educational level	primary school	26 (3.35%)
	middle school	108 (13.92%)
	high school or technical secondary school	139 (17.91%)
	junior college	213 (27.45%)
	bachelor degree or higher	290 (37.37%)
Type of puerpera	primiparaes	353 (45.49%)
	multiparas	423 (54.51%)
Type of birth	natural birth	481 (61.98%)
	cesarean birth	295 (38.02%)

### Correlations of the investigated variables

Pearson’s correlation test was used to conduct correlational analysis of each variable. Table 2 shows that postpartum negative life events, neuroticism, experiential avoidance, and PPD were all significantly correlated.

**Table 2:** Descriptive analysis and correlations

	M	SD	1	2	3	4
1. PMLE	79.988	25.979	1			
2. Neuroticism	31.572	9.046	0.493**	1		
3. EA	20.178	10.154	0.674**	0.552**	1	
4. PPD	9.102	5.117	0.546**	0.575**	0.560**	1

*Note* PNLE = Postpartum negative life events; EA = Experiential avoidance; PPD = Postpartum depression. **p < 0.01

### Mediation analysis

After controlling for age and education level, Hayes’ SPSS Process 2.15 Model 4 was used to test the mediating effect of neuroticism between postpartum negative life events and PPD. The results are shown in Table 3. In the mediating model, neuroticism had a significant positive effect on PPD (*β* = 0.388, *t* = 12.378, *p* < 0.001), postpartum negative life events had a significant direct effect on PPD (*β* = 0.340, *t* = 10.841, *p* < 0.001), and postpartum negative life events had a significant indirect effect on PPD (*β* = 0.188, *t* = 6.383, *p* < 0.001), with 95% CI [0.133, 0.253]. Indirect effect accounted for 35.61% of the total effect. These results indicate that neuroticism partially mediated the relationship between postpartum negative life events and PPD.

**Table 3:** Mediation analysis using SPSS process model 4

Outcome variable	Independent variables		β	SE	t	p
	Control variables	Predictors				
Neuroticism	Age		-0.008	0.007	-1.189	0.235
	Education level		-0.077	0.028	-2.748	0.006
		PNLE	0.485	0.031	15.417***	< 0.001
*R*^*2*^ = 0.254						
*F* = 87.225***						
PPD	Age		-0.017	0.006	-2.933**	0.004
	Education level		-0.067	0.025	-2.745**	0.006
		Neuroticism	0.388	0.031	12.378***	< 0.001
		PNLE	0.340	0.031	10.841***	< 0.001
*R*^*2*^ = 0.435						
*F* = 147.939***						

*Note* PNLE = Postpartum negative life events; EA = Experiential avoidance; PPD = Postpartum depression. p** < 0.01, *** *p* < 0.001

### Moderated mediation analysis

After the mediation test, Hayes’ SPSS process 2.15 Model 15 was used to test the moderated mediation effect. The results are shown in Table 4. Neuroticism significantly positively predicted postpartum depression (*β* = 0.287, *t* = 8.745, *p* < 0.001, 95% CI [0.223, 0.351]), and the interaction between neuroticism and psychological flexibility was significant in predicting PPD (*β* = 0.058, *t* = 2.410, *p* < 0.05, 95% CI [0.011, 0.105]), suggesting that psychological flexibility plays a moderating role between neuroticism and PPD. Postpartum negative life events significantly positively predicted PPD (*β* = 0.188, *t* = 5.141, *p* < 0.001, 95% CI [0.116, 0.260]), and the interaction term between postpartum negative life events and experiential avoidance was significant in predicting PPD (*β* = 0.080, *t* = 3.108, *p* < 0.01, 95% CI [0.030, 0.131]), indicating that psychological flexibility plays a moderating role in the influence path of postpartum negative life events on PPD. These results show that the moderated mediation effect is valid.

**Fig. 2:**
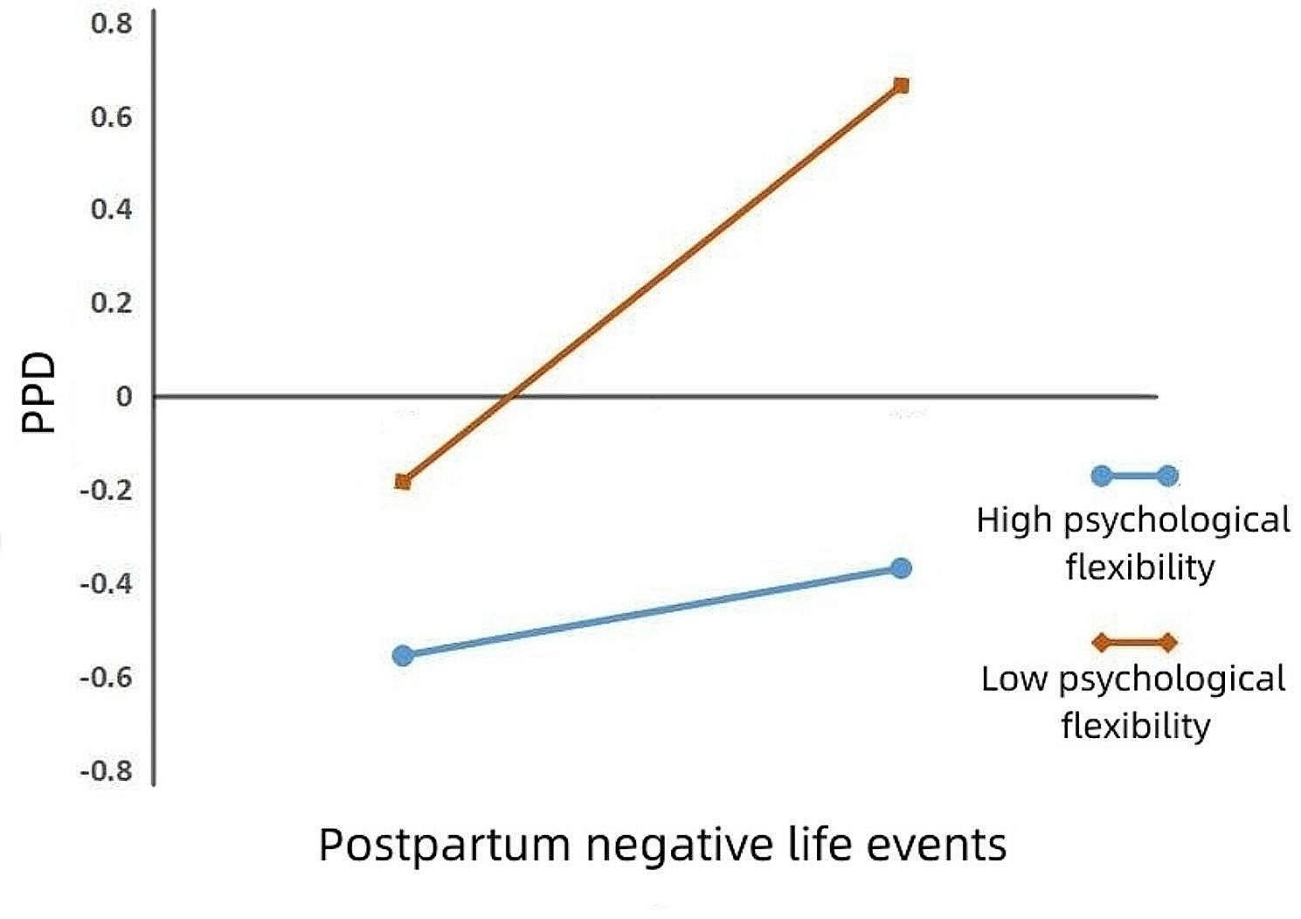
The moderating role of psychological flexibility in the process of postpartum negative life events affecting PPD

**Fig. 3:**
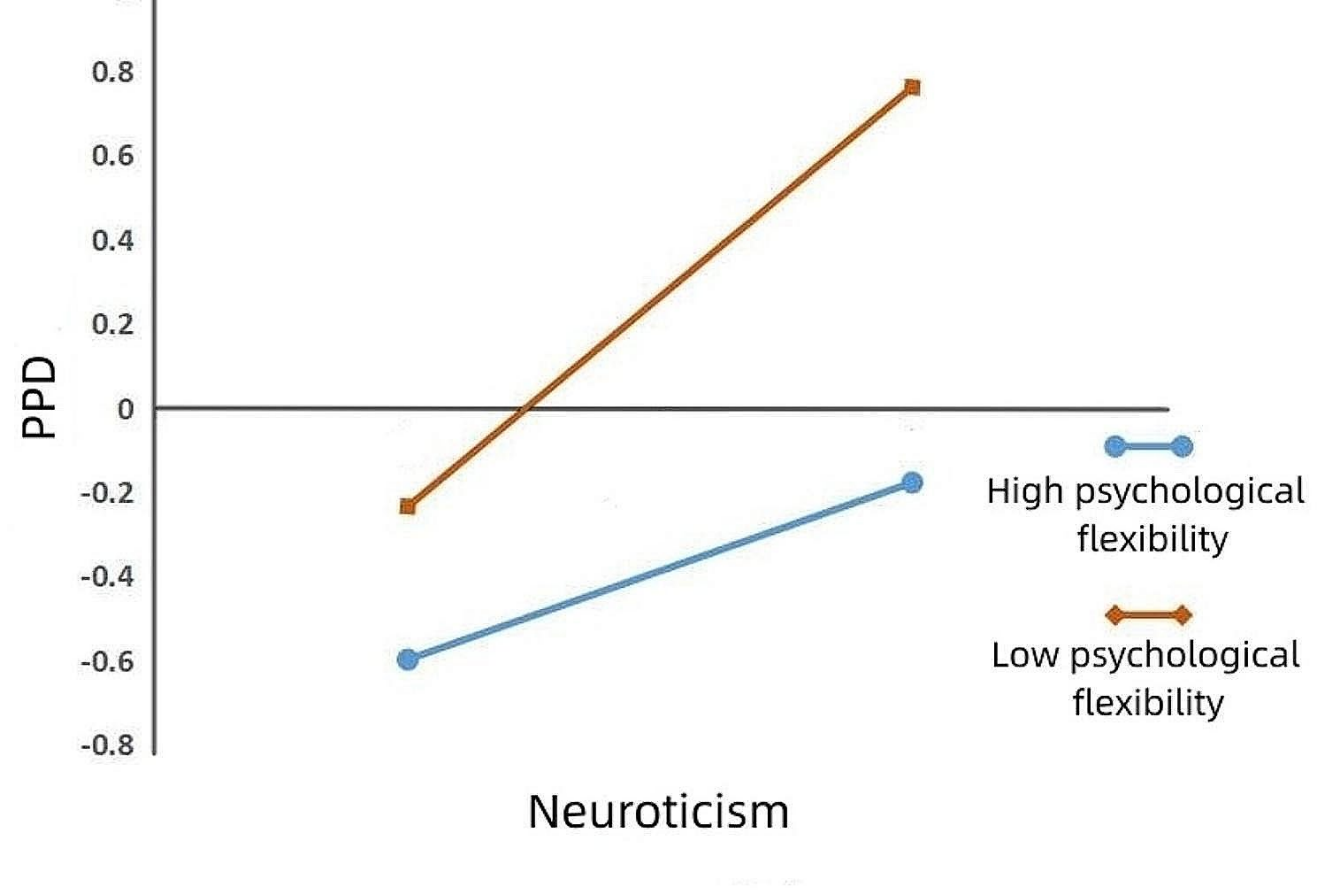
The moderating role of psychological flexibility in the process of neuroticism affecting PPD

To further understand the moderating effect of psychological flexibility, we divided experiential avoidance into high and low groups, using plus or minus one standard deviation. A simple slope test was used to investigate the moderating effect of psychological flexibility. The results showed that negative life events significantly predicted PPD in parturients with low psychological flexibility (i.e., high experiential avoidance; *β*_simple_ = 0.424, *t* = 10.383, *p* < 0.001, 95% CI [0.344, 0.504]). For parturients with high psychological flexibility (i.e., low experiential avoidance), negative life events did not predict PPD (*β*_simple_ = 0.094, *t* = 1.959, *p* > 0.05, 95% CI [-0.0002, 0.188]; see Fig. 2). This indicates that a high level of psychological flexibility can effectively alleviate the effects of negative life events on PPD. These results also show that neuroticism positively predicted PPD in parturients with low psychological flexibility (i.e., high experiential avoidance; *β*_simple_ = 0.469, *t* = 12.949, *p* < 0.001, 95% CI [0.398, 0.540]). Neuroticism also significantly positively predicted PPD in parturients with high psychological flexibility (i.e., low experiential avoidance; *β*_simple_ = 0.240, *t* = 5.758, *p* < 0.001, 95% CI [0.158, 0.322]), but the predictive effect was reduced (see Fig. 3). This suggests that high levels of psychological flexibility can effectively mitigate the effect of neuroticism on PPD.

**Table 4:** Moderated mediation analysis by process model 15

Outcome variable	Independent variables		β	SE	t	p
	Control variables	Predictors				
Neuroticism	Age		-0.008	0.007	-1.189	0.235
	Education level		-0.077	0.028	-2.748	0.006
		PNLE	0.485	0.031	15.417***	< 0.001
*R*^*2*^ = 0.254						
*F* = 87.225***						
PPD	Age		-0.018	0.006	-3.305**	0.001
	Education level		-0.056	0.024	-2.377*	0.018
		Neuroticism	0.287	0.033	8.745***	< 0.001
		PNLE	0.188	0.037	5.141***	< 0.001
		EA	0.230	0.038	6.060***	< 0.001
		Neuroticism × EA	0.058	0.024	2.410*	0.016
		PNLE × EA	0.080	0.026	3.108**	0.002
*R*^*2*^ = 0.481						
*F* = 101.214***						

*Note* PNLE = Postpartum negative life events; EA = Experiential avoidance; PPD = Postpartum depression. *p < 0.05, **p < 0.01, ***p < 0.001

## Discussion

The current study integrated postpartum negative life events, neuroticism, psychological flexibility, and PPD into one model. Using this moderated mediation model, we examine the mediating effect of neuroticism and the moderating effect of mental flexibility on PPD. Our results suggest that postpartum negative life events can positively predict PPD in Chinese parturients, and neuroticism plays a mediating role in this relationship. Psychological flexibility not only moderates the relationship between postpartum negative life events and PPD, but also moderates the relationship between neuroticism and PPD.

### The direct impact on PPD

The results show that postpartum negative life events are significantly positively correlated with PPD and neuroticism, neuroticism is significantly positively correlated with PPD, experiential avoidance is significantly negatively correlated with PPD, and postpartum negative life events can positively predict PPD, which is consistent with the findings of previous studies [12, 37, 50]. Hypothesis 1 has been verified. According to diathesis–stress model, stressors are antecedent variables that can cause mental disorders [10]. Postpartum negative life events, such as a lack of social support, postpartum complications, obstruction of normal life, economic pressure, marital discord, lack of parenting experience, and lack of family support are the main stressors parturients experience. Faced with these stressors, parturients are prone to activating dysfunctional beliefs and producing more biased interpretations, increased negative thoughts, and suffering from negative emotional experiences, thus leading to depression [11].

### The mediating role of neuroticism

The mediating effect test found that neuroticism played a partial mediating role in the relationship between postpartum negative life events and PPD. Postpartum negative life events can not only indirectly affect PPD through influencing neuroticism, but also directly affect PPD, which is consistent with findings of previous studies focused on college students and adolescents [23, 24]. Hypothesis 2 is supported. According to the diathesis–stress model, negative life events as stressors and neuroticism as a vulnerability factor are both important risk factors for PPD [10]. The predisposition model also holds that personality traits and stressful life events play a significant role in the development and processing model of depression [16]. Previous studies have confirmed that neuroticism, as the strongest personality trait predictor of PPD [22], plays an important role in predicting PPD together with life events [24]. Individuals with a high level of neuroticism tend to have a higher baseline level of negative emotions, higher sensitivity to external stimuli, and an increased attentional bias toward threatening information [18, 19]. They are more likely to experience negative emotions such as fear, sadness, shame, anger, or disgust, and will have lower abilities to cope with pressure or regulate their emotions, and high degrees of negative thinking and life pressure. They are more likely to be depressed than people with a low level of neuroticism [51, 52]. At the same time, although personality characteristics have a certain stability, they also have a degree of plasticity, and can be affected by environmental pressure. The dynamic predisposition model suggests that life events stress and other factors can affect individual personality traits, both of which are predictive variables of depression [26, 27]. Previous studies have consistently found that stressful life events predict a subsequent increase in neuroticism [28, 29, 53]. Prior research distinguishes three possible sources of variance for neuroticism: an allegedly stable set-point-neuroticism component representing the general trait-level of neuroticism, a transient state-deviation component which changes in response to life events and current psychopathology, and an error component [53]. In the short term, negative life events can lead to transient state-deviation in neuroticism due to changes in self-perception. This change in neuroticism may be short-term, and when negative life events decrease, the level of neuroticism may return to set point. In the long term, persistent negative life events can cause stress in individuals, and those who are exposed to long-term stress and cannot cope effectively are more prone to frustration, through which adverse coping styles are strengthened, cognitive structures are changed, and the levels of neuroticism (as a general trait) are also increased correspondingly. Therefore, postpartum negative life events can not only directly affect PPD, but they can also lead to an increase in one’s level of neuroticism, thereby affecting PPD.

#### The moderating role of psychological flexibility

This study also found that psychological flexibility plays a moderating role in both the direct path and the second half of the indirect path of the mediation model, supporting Hypothesis 3. According to both the diathesis–stress and the psychological flexibility models, psychological flexibility is an important protective factor for mental health [15, 32], comprising six core components: acceptance, defusion, self as context, contact with the present moment, values, and committed action, all of which help regulate mental health and improve function [33].

According to ACT, in the face of negative life events as a stressor, parturients with low psychological flexibility are more inclined to attach themselves to the conceptualized self, hold inflexible contact with the present moment, underestimate their own coping ability, and overestimate the true difficulties of negative life events, making it difficult for them to take effective action to deal with these events and thus enhancing the impact of the negative life events on PPD [33]. In contrast, parturients with high psychological flexibility are better able to maintain self-awareness through mindfulness and other means, holding a more flexible attention to the present moment, and engaging in positive cognitive reappraisal to redefine and evaluate negative life events [33, 54]. They can then take meaningful actions to cope, thus alleviating the risk of depression caused by negative life events after childbirth.

Neurotic individuals have higher sensitivity to stimuli as well as higher baseline levels of negative emotions [55]. Neurotic parturients experience greater psychological pressure, increased unrealistic thoughts, excessive demands and impulsivity, are more inclined to evaluate social support, family economic status, and newborn feeding negatively, and are more prone to anger, anxiety, depression, and other negative experiences [21]. In the face of negative experiences brought about by a neurotic personality, parturients with low psychological flexibility often adopt more experiential avoidance to deal with them, that is, they are unwilling to keep in touch with their negative experiences (e.g., physical feelings, thoughts, memories, emotions) and instead try to control or avoid them [32]. Although experiential avoidance appears to have a short-term protective effect, in the long term it can increase the frequency of negative experiences (that is, unprocessed and ignored negative experiences will reoccur more frequently) and even lead to rumination, which can produce, maintain, or exacerbate depressive symptoms [33, 56]. However, parturients with high psychological flexibility are more likely to adopt acceptance and mindfulness, and be able to take meaningful actions to cope with their negative experiences so as to interrupt the mechanism of depression caused by experiential avoidance, thereby relieving the risk of depression caused by neuroticism [36, 57].

### Implications and limitations

The current study combined the diathesis–stress model and ACT to explore the influence mechanism of postpartum negative life events on PPD, and our findings have certain implications for the prevention and intervention of PPD. First, it is important to pay special attention to the life events experienced by parturients, and to aid them in analyzing and resolving their difficulties so as to reduce the impact of these events on depression. Second, special attention should be paid to parturients with a neurotic personality, offering relevant education regarding mental health to help them to alleviate the adverse effects of their neurotic personality as much as possible. Finally, we acknowledge that postpartum negative life events have a certain degree of uncontrollability, while a neurotic personality has a certain degree of stability, and both are difficult to change through intervention. However, psychological flexibility can effectively alleviate the effects of postpartum negative life events and neuroticism on PPD. Therefore, improving maternal psychological flexibility is possible. Specifically, parturients should be taught some ACT skills (such as acceptance, flexible focus on the present, mindfulness, self-as-context, commitment to valuable actions, etc.), so that parturients accept postpartum negative life events, and change according to the situation, constantly adjust their thinking, imagination and memory functions, to avoid falling into the vicious circle of negative automated thoughts. It also enables parturients to allocate more attention and resources to what is important and to act on their own and their family’s life goals.

Although the current study does provide some enlightenment regarding the prevention and intervention of PPD, there are nonetheless some limitations. First, The vulnerability factors for PPD also include biological factors. Current studies have only studied the mechanism of PPD from the perspective of social and psychological factors; biological factors should be taken into account in future research. Second, the current study adopted a cross-sectional research design; the moderated mediation model proposed in the current study should be tested further through longitudinal research in the future. Third, some researchers have pointed out that AAQ-II has some shortcomings. AAQ-II has variable reliability and discriminant validity [46]. To infer psychological flexibility from psychological inflexibility will have some shortcomings [58]. Therefore, more effective instruments for measuring psychological flexibility, such as the Personalized Psychological Flexibility Index (PPFI), should be considered in future studies. Finally, the samples selected in current study were from three hospitals in three provinces in China, so the research results may have certain limitations; future research should expand the scope of investigation.

## Conclusion

The main conclusions of the current study are as follows: postpartum negative life events can positively predict postpartum depression. Neuroticism plays a mediating role between postpartum negative life events and postpartum depressive symptoms. Psychological flexibility has a moderating effect on the direct path of postpartum negative life events affecting postpartum depression, as well as a moderating effect in the second half of the mediating effect of neuroticism on postpartum negative life events and PPD.

## Data Availability

The datasets generated for this study are available upon request to the corresponding author.
